# The Effects of Male Seminal Fluid Proteins on Gut/Gonad Interactions in Drosophila

**DOI:** 10.3390/insects13070623

**Published:** 2022-07-13

**Authors:** Melissa A. White, Mariana F. Wolfner

**Affiliations:** 1 Department of Molecular Biology and Genetics, Cornell University, Ithaca, NY 14853, USA; 2 Baker Institute for Animal Health, Cornell University, Ithaca, NY 14853, USA

**Keywords:** inter-organ communication, signaling, post-mating response, sex peptide

## Abstract

**Simple Summary:**

The functions of organ systems must be coordinated for physiological homeostasis to occur. For example, after mating, coordination between insect digestive and reproductive systems is needed to ensure adequate nutrition for efficient egg/progeny production, and, conversely, to attune egg production levels to nutrient availability. Recent studies of Drosophila have revealed much about the post-mating changes in female reproductive tract function and in gut homeostasis, and the induction of these changes by male seminal proteins. Interesting regulatory connections between the organ systems and their responses have come to light in those studies. We have gathered these data into a single network schematic of the signaling events that operate within and between the reproductive and digestive systems downstream of seminal fluid proteins, summarizing current knowledge of the crosstalk between the systems and raising open questions for future study.

**Abstract:**

Mating initiates broad physiological changes encompassing multiple organ systems in females. Elucidating the complex inter- and intra-organ signaling events that coordinate these physiological changes is an important goal in the field of reproductive biology. Further characterization of these complex molecular and physiological interactions is key to understanding how females meet the energetic demands of offspring production. Many recent studies of the fruit fly, *Drosophila melanogaster*, have described the mechanisms of post-mating changes within the female reproductive tract and digestive system. Additionally, other studies have described post-mating signaling crosstalk between these systems. Interestingly, male seminal fluid proteins have been linked to post-mating responses within the female reproductive tract and gut, and to signaling events between the two organ systems. However, information about the hormonal and neuronal signaling pathways underlying the post-mating signaling events within and between the reproductive tract and digestive systems that are triggered by seminal fluid proteins has yet to be combined into a single view. In this article, we summarize and integrate these studies into a single “network schematic” of the known signaling events within and between the reproductive and digestive systems downstream of male seminal fluid proteins. This synthesis also draws attention to the incomplete parts of these pathways, so that outstanding questions may be addressed in future studies.

## 1. Introduction

Throughout the animal kingdom, female nutrition is connected to reproductive output. Females must consume enough appropriate nutrients to energetically support pregnancy (in mammals) [1] or the production of large numbers of eggs (as in insects) [2]. The energetic costs of offspring production are thus demanding on the female digestive system, which must efficiently process food and absorb nutrients to sustain both somatic homeostasis and reproduction. Crosstalk between the reproductive and digestive systems, which coordinates post-mating physiological changes, is critical for reproductive success. This crosstalk can occur via the nervous system relaying signals about the female’s reproductive state [3,4,5], via hormones [6,7,8,9,10], and via intra- and inter-organ signals from, or acting on, different types of cells in the female reproductive tract or gut [11,12,13,14]. 

**Figure 1:**
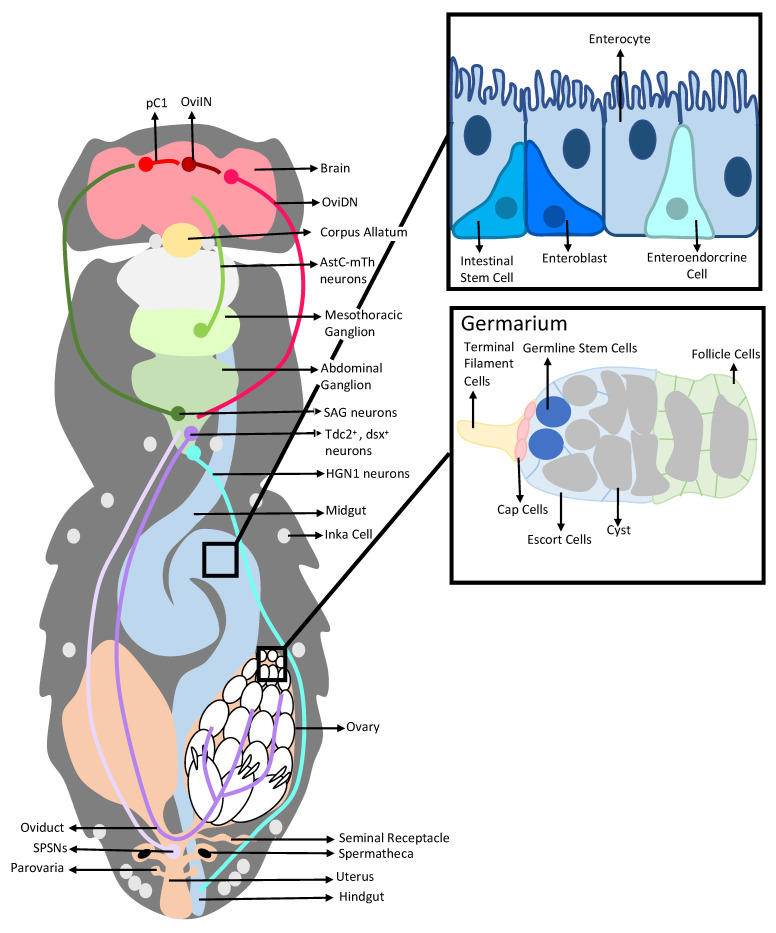
Anatomical diagram of the organs and cell types of *Drosophila melanogaster* females that are involved in post-mating responses triggered by seminal proteins in the reproductive, digestive and nervous systems (not to scale). Figure elements are adapted from [33,34,35].

The genetic tractability of *Drosophila melanogaster* makes it an excellent system for dissecting how changes within the digestive and reproductive systems, as well as crosstalk between these two organ systems, can affect offspring production. After mating, *Drosophila melanogaster* females undergo a suite of physiological and behavioral changes that transition the female from a virgin state to a highly fecund mated state. Collectively, these changes are referred to as the post-mating response (PMR). In the female reproductive tract (Figure 1), the PMR includes increasing egg production [15,16,17], ovulation [18,19], oviposition [20], and mediation of the storage and regulated release of sperm [21,22,23,24]. In the digestive system (Figure 1), the PMR increases the efficiency of digestion by modifying gut size, gene expression, and function [11,14,25,26]. Genetic, molecular, physiological, and neurobiological experiments have shown that these changes are mediated by a variety of molecules or signals, including seminal fluid proteins (SFPs) from the male [27], hormones (juvenile hormone (JH) and ecdysone (20E)) [7,9,10,15], neuromodulators (such as octopamine (OA)) [13,28,29,30,31], and neural circuit activity in the female [3,4,5,10,13,20,25,32]. These actors can trigger post-mating responses within the reproductive and digestive systems, and mediate signaling between organ systems that may both sustain and fine-tune post-mating physiological changes [11,12]. These networks and pathways are only just coming to light. 

**Figure 2:**
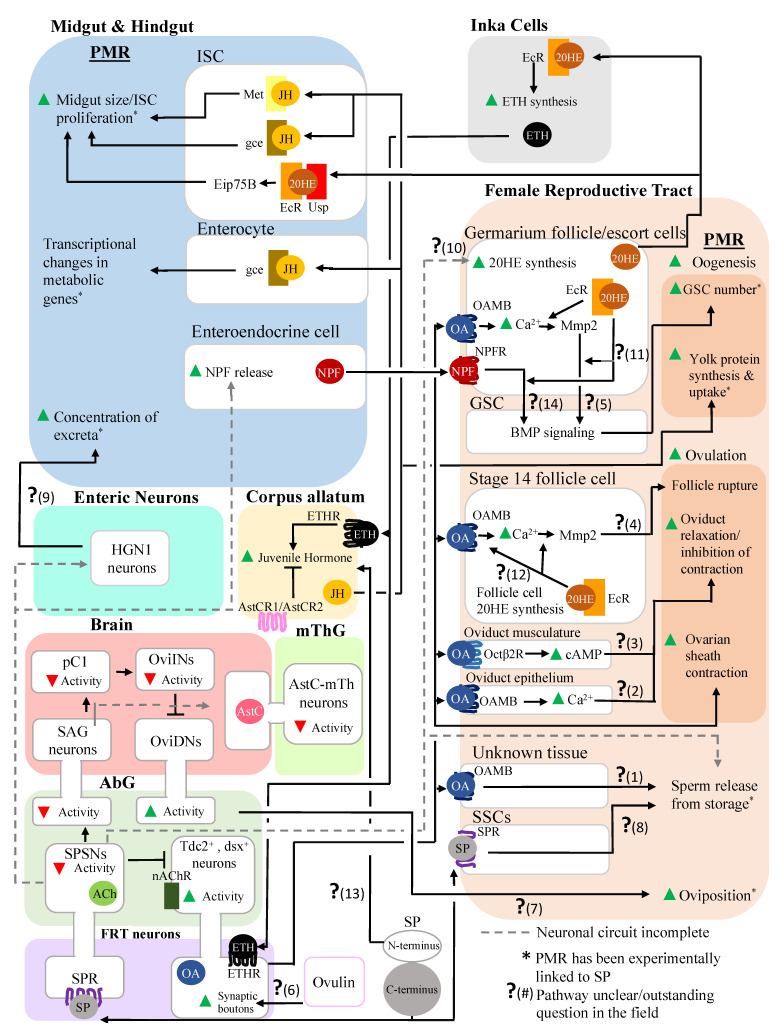
Network schematic of the signaling events within and between the digestive, nervous, and reproductive systems in response to seminal fluid proteins received during mating. Tissues are colored as in Figure 1. 20HE: 20-hydroxyecdysone, AbG: abdominal ganglion, Ach: acetylcholine, AstC: allatostatin C, AstCR1: allatostatin C receptor 1, AstCR2: allatostatin C receptor 2, cAMP: cyclic adenosine monophosphate, EcR: ecdysone receptor, ETH: ecdysis-triggering hormone, ETHR: ecdysis-triggering hormone receptor, FRT: female reproductive tract, Gce: germ cell expressed (a juvenile hormone receptor [36]), GSC: germline stem cell, ISC: intestinal stem cell, JH: juvenile hormone, Met: methoprene-tolerant (a juvenile hormone receptor [37]), Mmp2: matrix metalloprotease 2, mThG: mesothoracic ganglion, nAChR: nicotinic acetylcholine receptor, NPF: neuropeptide F, NPFR: neuropeptide F receptor, OA: octopamine, OAMB: octopamine receptor in mushroom bodies, Octβ2R: octopamine β2 receptor, OviDN: oviposition descending neurons, OviIN: oviposition inhibitory neurons, PMR: post-mating response, SP: sex peptide, SPR: sex peptide receptor, SPSN: sex peptide sensory neuron, SSCs: spermathecal secretory cells, Usp: ultraspiracle (part of the 20E receptor [38]).

Here, we present and briefly discuss a “network schematic” (Figure 2) that summarizes the current state of knowledge in the field. Given the complex nature of the post-mating interactions within and between organ systems, we found that having such a schematic helped us to systematize our own thoughts, and we hope that it will be useful to other researchers as well. We refer readers to the references section of this paper for a deeper analysis of each node of the schematic, and note where further investigation of a particular interaction is needed. We hope that by documenting and diagramming the regulatory connections among reproduction/mating, the reproductive tract, and the digestive system, this article will stimulate future research into the mechanism of reproductive–digestive cooperation. 

## 2. Interactions between a Seminal Protein and the Nervous System Affect the Reproductive and Digestive Systems

A key step in the coordination of post-mating responses between the reproductive and digestive systems is the initiation of responses in both systems by receipt of the seminal fluid protein, sex peptide (SP), during mating. This 36-amino-acid peptide triggers transitions from a virgin to a mated state [6,7,10,11,13,14,16,17,39,40,41]. SP is initially transferred to females within the seminal fluid. Then, in the female reproductive tract, SP binds to the sperm by its N-terminus and is thereby retained in the female’s sperm storage organs (Figure 1). Gradually, the C-terminus of SP, which induces the PMR in both the gut and reproductive tracts, is released from the sperm by trypsin cleavage [42]. The free C-terminus of SP then binds to the sex peptide receptor (SPR), a G-protein coupled receptor (GPCR) located in the sperm storage organs and on the SP sensory neurons (SPSNs) in the uterus (Figure 1) [4,43,44]. SP binding to the SPR on the SPSNs silences the SPSNs’ activity, which in turn affects the activity of downstream neuronal circuits [3,5,10,13,20,32,45].

### 2.1. Sex Peptide, SP-SPSNs, and the Female Reproductive Tract

Changes in the activity of neurons that innervate the reproductive tract are a key means by which PMRs are initiated. One set of neurons affected by SPSN activity are the octopaminergic *Tdc2^+^*, *dsx^+^* neurons that project to the female reproductive tract from the abdominal ganglion (Figure 1) [13,46,47]. SP-induced silencing of the SPSNs after mating attenuates their cholinergic inhibition of the female reproductive tract’s octopaminergic neurons [13]. The subsequent increase in OA within the female reproductive tract triggers PMRs, including an increase in germline stem cell (GSC) numbers via the actions of the OA receptor OAMB, as well as Ca^2+^ increases and matrix metalloproteinase 2 (Mmp2) activity in a subset of somatic cells in the germarium of the ovary (the site of the GSCs and early oogenesis (Figure 1)), and BMP signaling in the GSCs themselves [13]. At later stages in egg production, OA stimulates follicle rupture, which releases the oocyte from its casing of follicle cells [48]. OA also modulates the contraction and relaxation of the reproductive tract musculature to move the oocyte out of the ovary and into the lateral oviduct during ovulation [8,19,49,50]. For example, OA relaxes the oviduct musculature while stimulating contraction of the ovary’s muscle sheath [8,19,49,50]. Additionally, OA modulates the release of sperm from the storage organs via OAMB in an unknown tissue (Figure 2) [22]. Thus, by triggering post-mating responses in multiple cell types within the female reproductive tract, OA coordinates high levels of egg production, ovulation, and fertilization [51]. Since SP is an upstream trigger for the disinhibition of reproductive tract *Tdc2^+^*, *dsx^+^* neuron activity [13], SP may lie upstream of some or all OA-induced PMR phenotypes in the reproductive tract. However, to date, only SP’s effect on post-mating GSC proliferation has been experimentally linked to *Tdc2^+^*, *dsx^+^* neuron activity [13]. An important direction for future work will be to establish whether SP and the SP–SPSN–OA axis lie upstream of the other OA-induced PMRs in the reproductive tract, as well as the downstream signaling pathways linking this axis to PMR phenotypes (question marks 1–5 in Figure 2). 

Interactions between the SP–SPSN–OA axis and ovulin, another seminal fluid protein that affects OA signaling, should also be examined [19]. Ovulin, a prohormone-like protein, stimulates ovulation by increasing the number of octopaminergic neuron synaptic boutons on the oviduct [18,19]. The identification of the ovulin receptor and the downstream pathways that lead to bouton outgrowth will be key to understanding ovulin’s mechanism of action (question mark 6 in Figure 2). Moreover, since both SP and ovulin increase the activity of OA neurons after mating, the relative contribution of each SFP to OA-induced PMRs should be examined. Since ovulin acts only within the first 24 h after mating, while SP’s effects persist for at least a week, it is possible that ovulin’s early stimulation of OA neuronal outgrowth primes the OA neurons for the prolonged increase in activity triggered by SP’s inhibition of the SPSNs [13,18,41,52]. 

SP–SPSN neuronal signaling also triggers physiological changes in the female reproductive tract via other neuronal circuits. An SP–SPR-regulated neuronal circuit containing the SPSNs, SAG neurons in the abdominal ganglion, pC1, and oviposition inhibitory (oviINs) neurons in the brain controls oviposition, in conjunction with oviposition descending neurons (oviDNs) that project from the brain to the abdominal ganglion (Figure 1) [20]. Specifically, silencing the SPSNs by SP–SPR binding in turn silences the SAG, pC1, and oviINs. This silencing of the oviINs disinhibits the oviDNs, thus stimulating oviposition [20]. Downstream events linking the oviDNs to oviposition are yet to be characterized (question mark 7 in Figure 2). 

SP–SPR signaling does not only trigger post-mating responses via the SPSNs. The SPR is also expressed in the oviduct and in the spermathecal secretory cells (SSCs) [4]. While the role of the SPR in the oviduct remains unknown, SPR expression in the SSCs is needed for the proper release of sperm from the storage organs [21]. The mechanism through which SSCs influence sperm release from the storage organs is unknown (question mark 8 in Figure 2), but this pathway may involve molecules secreted from SSCs. Interestingly, SP–SPSN neuronal signaling is also required for efficient sperm release from the storage organs [21]. Thus, SP effects efficient sperm release from the storage organs both directly via the SPR on the SSCs, and indirectly via neuronal signaling. This further demonstrates how SP triggers PMRs by activating synergistic, parallel pathways.

### 2.2. Sex Peptide’s Interaction with the Nervous System Also Modulates Gut Function and Physiology

SP–SPSN neuronal signaling also affects multiple aspects of intestinal physiology. SP–SPSN neuronal signaling plays a key role in remodeling intestinal physiology to cope with the nutritional demands of egg production [25]. SP and neuronal SPR are also required for increasing nutrient absorption and creating more concentrated excreta by modulating intestinal fluid homeostasis [25,53]. Though these SP-induced changes in intestinal fluid balance have been linked to *HGN1-GAL4*-positive neurons in the hindgut (Figure 1), the neural circuit linking SP, and potentially the SPSNs, to the *HGN1-GAL4* neurons remains unknown [25]. SP–SPSN neuronal signaling also lies upstream of the neuropeptide F (NPF) release from midgut enteroendocrine cells (EEs) [12]. Additionally, SP and its receptor, SPR, are needed for post-mating gut growth [27]. It is not yet known whether SPSN neural activity regulates the increase in post-mating gut size [27]. Future work should further establish the neuronal circuits linking SP and the SPSNs to enteric neuron activity and identify the signaling molecules these neurons utilize to alter excreta composition (question mark 9 in Figure 2). 

## 3. Post-Mating Hormonal Changes Induced by SP also Affect Both the Reproductive and the Digestive Systems

Another important way in which SP alters digestive and reproductive tract physiology, as well as crosstalk between the two, is by manipulating the ecdysone–ecdysis-triggering hormone–juvenile hormone (20E–ETH–JH) axis. Under normal conditions, 20E stimulates ETH expression in Inka cells (Figure 1) and ETHR expression in target tissues [9]. ETH then binds to the ETHR on the corpus allatum (a major site of synthesis of JH (Figure 1)), stimulating an increase in systemic JH levels [9]. This hormonal axis can also interact with OA signaling, as ETH can stimulate OA release in the female reproductive tract by binding to the ETHR on reproductive tract octopaminergic neurons [8]. SP can trigger hormonal changes directly by binding to target tissues involved in hormone synthesis [6], or indirectly via SP–SPSN neuronal signaling [7,10,17].

### 3.1. Alteration of JH and 20E Levels Mediated by SP Stimulates Female Reproductive Physiology

SP stimulates 20E synthesis in a subset of somatic cells in the germarium after mating [7]. The stimulation of 20E synthesis is mediated by SP–SPSN neuronal signaling, although the mechanistic details linking the SPSNs to 20E are not yet known (question mark 10 in Figure 2) [7,17]. The 20E binds to the ecdysone receptor (EcR) in germarium somatic cells, where it is required for the post-mating increase in the number of GSCs [17]. At a later stage in oogenesis, 20E produced in stage-14 follicle cells is required for follicle rupture during ovulation [54]. However, to date, 20E synthesis in stage-14 follicle cells has not been linked to SP–SPSN signaling. Interestingly, there is an interaction between 20E and OA signaling within the female reproductive tract, as 20E–EcR signaling is required for OA-induced stimulation of follicle rupture and for GSC proliferation via Ca^2+^ and Mmp2. However, again, the details of this interaction are unknown (question marks 11 and 12 in Figure 2) [7,13,17,48,54]. The abovementioned SP-mediated increase in 20E can in turn affect OA levels via ETH [8]. Thus, it is possible that SP may be influencing OA levels both hormonally via 20E and ETH, and neuronally via the SP–SPSN–OA axis. Further study is required to disentangle the relative contributions of each pathway to the SP-induced changes in OA signaling. Moreover, given that SP increases 20E synthesis in germarium somatic cells and the activity of octopaminergic neurons, it will be intriguing to determine whether SP affects follicle rupture in mature oocytes, a process that is known to require 20E and OA but has not yet been linked to SP [48,54].

Higher levels of systemic JH after mating are essential for post-mating increases in egg production [15,16]. SP increases JH levels both directly and indirectly. The N-terminus of SP acts directly on the corpus allatum to stimulate JH release via a currently-unknown mechanism (question mark 13 in Figure 2) [6,55,56]. Silencing of the SP-SPSNs and SAG neurons upon mating also silences the AstC-mTh neurons, which inhibit JH synthesis in the corpus allatum [10]. Since a free N-terminus of SP is only transiently present within the female [41], it is possible that this portion of SP leads to a transient increase in JH post-mating, with the maintenance of high JH synthesis requiring SP–SPSN signaling and subsequent silencing of the AstC-mTh neurons [10]. It is also possible that SP may indirectly stimulate JH synthesis via 20E and ETH [9]. The rise in systemic JH after mating stimulates egg production by increasing yolk protein synthesis, increasing yolk protein uptake by developing oocytes and allowing oogenesis to progress beyond the checkpoint at stage 9 [15,16]. 

### 3.2. Alteration of JH and 20E Levels Mediated by SP Affects the Digestive System

JH from the corpus allatum acts directly on the digestive tract’s intestinal stem cells (ISCs) via the JH receptors Met and Gce, initiating growth of the midgut that, in turn, increases the digestive capacity of the gut in the mated female [14]. In enterocytes, JH acts via its receptor, Gce, to enhance lipid metabolism. SP has also been linked to JH-induced changes in midgut size, and to changes in the post-mating midgut transcriptome [26]. Specifically, SP triggers an upregulation of genes involved in proteolysis and a downregulation of genes involved in carbohydrate metabolism, which may reflect a metabolic shift in favor of protein digestion after mating [26]. 

20E can also trigger post-mating ISC proliferation and gut growth [11]. Currently, the relative roles of JH and 20E in post-mating gut growth remain poorly understood. However, since SP lies upstream of post-mating increases in both JH and 20E, it is possible that SP may influence gut growth through either or both hormones. Additionally, as both the JH and 20E receptors are transcription factors [45,57,58], it is possible that either JH or 20E, or both JH and 20E, contribute to the SP-induced changes in the midgut transcriptome [50,51,52]. 

## 4. Inter-organ Signaling Occurs between the Reproductive and Digestive Systems in Mated Females, Enabling Coordination of the Responses of These Systems

In addition to the coordination afforded by the triggering of relevant PMRs in the digestive and reproductive tracts by the same seminal protein (SP) and its downstream effectors, these organs’ responses are coordinated by signaling crosstalk between the systems. For example, the 20E made in germarium somatic cells stimulates both post-mating ISC proliferation and gut growth [11,17]. Thus, 20E derived from germarium somatic cells simultaneously stimulates GSC proliferation—and thus egg numbers—and changes midgut physiology to increase nutrient digestion and absorption, thus fostering the development of those eggs. Conversely, the gut produces signals that regulate oogenesis. For example, intestinal EEs release NPF, which triggers GSC proliferation by binding to its receptor, NPFR, on germarium somatic cells [12]. Analogous to the mechanism by which OA affects the proliferation of the GSCs, NPF–NPFR signaling also regulates BMP signaling in the GSCs, triggering self-renewal [12,13]. Interestingly, and also analogous to OA’s effect on GSCs, NPF’s effect on GSCs depends on 20E; however, how 20E affects NPF signaling is not yet understood (question mark 14 in Figure 2). Coming full circle, we note that the post-mating signaling events that contribute to post-mating GSC proliferation (NPF release from EEs, increased *Tdc2^+^*, *dsx^+^* neuron activity, and increased 20E synthesis in germarium somatic cells) all lie downstream of SP. Thus, signaling pathways downstream of SP can alter signaling both within and between organ systems to coordinate the female PMR.

## 5. Conclusions

It is our hope that the network schematic that we provide here can help to organize current knowledge in the field and to provide a framework for making predictions and designing experiments. As new information about the questions we have raised comes to light, or with the identification of entirely new regulators or interactions, the schematic can be revised to update the view of the post-mating signaling events in or between the reproductive and digestive systems. 

## Data Availability

Not applicable.
